# The traits of “trait ecologists”: An analysis of the use of trait and functional trait terminology

**DOI:** 10.1002/ece3.8321

**Published:** 2021-11-11

**Authors:** Samantha K. Dawson, Carlos Pérez Carmona, Manuela González‐Suárez, Mari Jönsson, Filipe Chichorro, Max Mallen‐Cooper, Yolanda Melero, Helen Moor, John P. Simaika, Alexander Bradley Duthie

**Affiliations:** ^1^ Swedish Species Information Centre Swedish University of Agricultural Sciences Uppsala Sweden; ^2^ Institute of Ecology and Earth Sciences University of Tartu Tartu Estonia; ^3^ Ecology and Evolutionary Biology School of Biological Sciences University of Reading Reading UK; ^4^ LIBRe – Laboratory for Integrative Biodiversity Research Finnish Museum of Natural History University of Helsinki Helsinki Finland; ^5^ Ecology and Evolution Research Centre School of Biological, Earth and Environmental Sciences University of New South Wales Sydney New South Wales Australia; ^6^ CREAF Cerdanyola del Vallès Spain; ^7^ Department of Water Resources and Ecosystems IHE Delft Institute for Water Education Delft The Netherlands; ^8^ Biological and Environmental Sciences University of Stirling Stirling Scotland

**Keywords:** community ecology, functional ecology, functional trait, trait

## Abstract

Trait and functional trait approaches have revolutionized ecology improving our understanding of community assembly, species coexistence, and biodiversity loss. Focusing on traits promotes comparability across spatial and organizational scales, but terms must be used consistently. While several papers have offered definitions, it remains unclear how ecologists operationalize “trait” and “functional trait” terms. Here, we evaluate how researchers and the published literatures use these terms and explore differences among subdisciplines and study systems (taxa and biome). By conducting both a survey and a literature review, we test the hypothesis that ecologists’ working definition of “trait” is adapted or altered when confronting the realities of collecting, analyzing and presenting data. From 486 survey responses and 712 reviewed papers, we identified inconsistencies in the understanding and use of terminology among researchers, but also limited inclusion of definitions within the published literature. Discrepancies were not explained by subdiscipline, system of study, or respondent characteristics, suggesting there could be an inconsistent understanding even among those working in related topics. Consistencies among survey responses included the use of morphological, phonological, and physiological traits. Previous studies have called for unification of terminology; yet, our study shows that proposed definitions are not consistently used or accepted. Sources of disagreement include trait heritability, defining and interpreting function, and dealing with organisms in which individuals are not clearly recognizable. We discuss and offer guidelines for overcoming these disagreements. The diversity of life on Earth means traits can represent different features that can be measured and reported in different ways, and thus, narrow definitions that work for one system will fail in others. We recommend ecologists embrace the breadth of biodiversity using a simplified definition of “trait” more consistent with its common use. Trait‐based approaches will be most powerful if we accept that traits are at least as diverse as trait ecologists.

## Graphical abstract

Trait and functional trait approaches are now widely used in ecology, but how ecologists operationalize trait terminology often is unclear. We use survey data and a literature review to evaluate how researchers across subdisciplines and study systems use functional trait terminology. We found inconsistencies in the use of “trait” and “functional trait” terms, but these inconsistencies were not explained by sub‐discipline or study system.

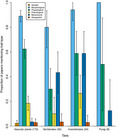

## 1 INTRODUCTION

Functional trait ecology originates from plant community ecology, and plants continue to dominate the functional trait research focus (Kraft et al., 2015; Kunstler et al., 2016; Shipley et al., 2016) with a growing body of literature using a functional trait approach in other organisms (e.g., Aguilar‐Trigueros et al., 2015; Dawson et al., 2019; Moretti et al., 2017). The emergence of trait‐based approaches has revolutionized the modeling of species distributions, population dynamics, species coexistence, and ecosystem functioning, particularly in the last two decades (Cadotte et al., 2015; González‐Suárez & Revilla, 2013; Laughlin & Messier, 2015; Moles, 2018; Schneider et al., 2019). Studying functional traits is key to answering important questions in ecology, allowing researchers to add trait information to species identities and investigate how the environment directly affects or is affected by different organisms based on functional traits (Gotzenberger et al., 2012; Legendre et al., 1997; Violle et al., 2007; Weiher & Keddy, 1995). This decoupling from reliance on species identities alone is one of the premises of functional trait approaches and allows them to be used even for groups in which species identity is hard to recognize (Carmona et al., 2016; Kraft et al., 2015; Messier et al., 2010). While functional trait approaches are touted as being applicable across taxa (Shipley et al., 2016), due to the plant‐centric background of trait‐based ecology, most trait theories and approaches have been constructed using plants as a model (e.g., Violle et al., 2007; Shipley et al., 2016; but see Gavel et al., 2016). The growing subset of functional ecology focused on other study organisms has led to incongruences in the use of concepts, definitions, and approaches for taxa that do not follow the same life‐form or organism concepts as plants.

Within the field of functional trait ecology, recent literature (Weiss & Ray, 2019) and discussions among peers (e.g., at meetings, workshops, and online blogs) suggests that there is disagreement regarding the proper use of the terms “trait” and “functional trait.” There is a range of definitions to draw upon, including the well‐known, but not fully overlapping or necessarily compatible definitions of Violle et al. (2007): “a trait is any morphological, physiological or phenological feature measurable at the individual level, from the cell to the whole‐organism level, without reference to the environment or any other level of organization”… “a functional trait as any M‐P‐P (morphological, physiological, or phenological) trait which impacts fitness indirectly via its effects on performance traits,” and McGill et al. (2006): “**Trait**: a well‐defined, measurable property of organisms, usually measured at the individual level and used comparatively across species. A **functional trait** is one that strongly influences organismal performance.” Functionality may be related to either the function of the individual (as per McGill et al., 2006; Violle et al., 2007) or function of ecosystem properties, for example, nutrient cycling, erosion, hydrology (Jax, 2005; Suding et al., 2008). Differences include the explicit mention of independence from environment, the use of organism vs. individual and the restriction of Violle et al. (2007) to a certain subset of traits (morphological, physiological, and phenological). Without a single accepted definition, researchers have applied the term in varying ways. Ambiguity around the use of the term “trait” or “functional trait” has been discussed by trait ecologists for many years (e.g., Violle et al., 2007). Yet, recently, several publications and commentary pieces have highlighted this as a continuing major shortcoming of functional trait ecology (Brodribb, 2017; McGill, 2015; Moles, 2018; Shipley et al., 2016). Some have offered new definitions or standardized sub‐terms to either encompass the broader way in which the term “trait” is currently applied (Schneider et al., 2019) or adapted the term for specific nonplant organisms (Bellwood et al., 2019; Dawson et al., 2019). One study went a step further and proposed “mechanistic” as opposed to “functional” traits (Brodribb, 2017). To support functional trait comparisons across taxa, Weiss and Ray (2019) advocate selecting functionally analogous traits that relate to community assembly processes. However, such cross‐taxa comparisons may not always be feasible or meaningful depending on the system and scale (Weiss & Ray, 2019), and trait approaches are still evolving, especially across environmental contexts and in understudied organism groups (Kissling et al., 2018). If traits and functional traits are to enable and enhance comparability between studies and analyses across spatial and organizational scales (Gavel et al., 2016; McGill et al., 2006; Shipley et al., 2016), there needs to be clarification and agreement in how ecologists define and use these terms (e.g., Garnier et al., 2017). However, no study has explicitly analyzed how ecologists define and use trait and functional trait terms, despite a huge increase in research applications across organisms and ecosystems over the last two decades.

To contribute to the evolving field of trait‐based ecology, at the 5th European Congress on Conservation Biology held from 12th to 15th of June 2018, we organized an open workshop on the use of functional traits in nonplant organisms. The presentations and discussions highlighted the disagreement among researchers on what categories make for acceptable “traits” and “functional traits.” Participants had a range of backgrounds and worked with different organisms including plants, fungi, insects, and vertebrates. From our conversations, we hypothesized that disagreements surrounding definitions of functional traits were likely to be based on researcher subdiscipline, taxon of interest, or biome studied. To test this hypothesis, first we conducted a post‐conference online survey to understand how functional trait ecologists conceptualize functional trait terminology, and second, we undertook a literature review to see how functional traits are presented in peer‐reviewed research publications. The purpose of the survey was to ascertain where there was agreement or divergence on trait conceptualization in relation to researcher subdiscipline, taxa of interest, or biome studied by a broader community of functional trait ecologists. By conducting both a survey and literature review, we tested the hypothesis that ecologists may have an opinion on traits that may be adapted or altered when confronting the realities of collecting, analyzing, and presenting data.

## 2 SURVEY ON THE INTERPRETATION AND APPLICATION OF TRAIT TERMINOLOGY

After the conference workshop, an anonymous online survey (Supplementary Information 1) was circulated to trait ecologists using Google Forms (https://www.google.com/forms/about/). The survey was publicly available from January to April 2019. We recruited participants through our network of contacts and diverse communicating platforms; for example, email lists, Twitter feeds. Participation was voluntary, and all participants were informed of the purpose of the survey and about their rights to refuse to answer any questions and to withdraw from participation at any time. While the survey was available for anyone to complete, we did ask participants to select categories of study area and research experience. Informed consent was obtained, anonymity and confidentiality were explicitly granted, and we did not include any information that could be used to identify individual respondents. All data were thus anonymized prior to analyses and are available (Supplementary Information 2).

In the survey, we asked a series of questions to capture the participants’ thoughts, interpretations and implementation of trait terminology. The first set of questions related directly to the participants’ understanding and use of traits and functional traits (Supplementary Information 1; Table S1.1), and the second set asked participants a series of self‐categorization questions (Supplementary Information 1; Table S1.2) so that we could partition answers by subdiscipline. Participant categorization included questions on researcher subdisciplines based on taxa studied, subfield(s), research focus, length of time they have been working with trait‐based approaches and (if applicable) the continents of their work institution and on which they conduct field work (Q9–Q17). The first set of questions Q1–Q6 covered aspects of “trait” definitions including relevance to different biological categories and scales, independence from the environment (a point of difference in several commonly used definitions), and heritability. Q7 and Q8 focused on “functional trait,” including conditions required to consider a trait as functional, and a classification exercise to mark listed traits as either functional traits or not. These last two questions were sourced from various “trait” definitions and “functional trait” papers respectively, so we could identify discrepancies between the published literature and ecologists’ understanding. All questions were carefully phrased to avoid prompting or biasing participants, and respondents selected from a range of options (e.g., strongly agree through to strongly disagree).

We hypothesized that variability among respondents’ opinions could be associated with different personal and professional backgrounds (e.g., experience, age, organism of specialization). We examined this question by creating a matrix of participant dissimilarity based on answers to survey questions using the R function “trova” (de Bello et al., 2013). This approach is frequently used in analyses of functional diversity, in which dissimilarity among organisms is estimated based on multiple traits. In our analysis, we considered participants as the organisms and their survey responses as “traits.” We then grouped respondents according to their personal and research features (subfield of research, studied taxa, biomes in which they work, and experience working with trait‐based approaches). For each of these groups, we created a series of virtual “communities” including all respondents who define themselves as being part of any of the categories defining that feature (e.g., “community ecologists” within the “subfield of research” feature). Some respondents considered themselves as being part of more than one group (e.g., respondents working in “community ecology” and “computational biology”), and thus could be represented in more than one group, just like a species can be part of different assemblages. We estimated the Rao index of diversity (de Bello et al., 2013; Carmona et al., 2016) that expresses the average functional dissimilarity between the organisms (in this case the researchers) composing a community (de Bello et al., 2016). The Rao index can also be estimated at different levels to partition functional diversity across scales (see de Bello et al., 2010 for details). We calculated Rao diversity for each of the communities (e.g., for each subfield of research) as well as for a pooled “ecosystem” that grouped all the different communities in each feature (e.g., grouping all subfields of research).

## 3 RESULTS OF TRAIT SURVEY

We received 486 responses to the survey. Most respondents worked in Europe (56%) or North America (20%), with 10% based in South America, 6% in Africa and <5% in Asia and Oceania. Respondents worked primarily on forest (35%), grassland (29%), or freshwater (11%) ecosystems, and defined their research in very diverse ways, with community ecology (17%), ecosystems ecology (11%), conservation science (11%), and population ecology (8%) being the most frequent answers. Most respondents had substantial experience with trait‐based approaches (81% of respondents had used traits in their work for more than 1 year, with ~20% having >10 years' experience); 18% of respondents had not worked with trait‐based approaches (Supplementary Information 3).

**FIGURE 1:**
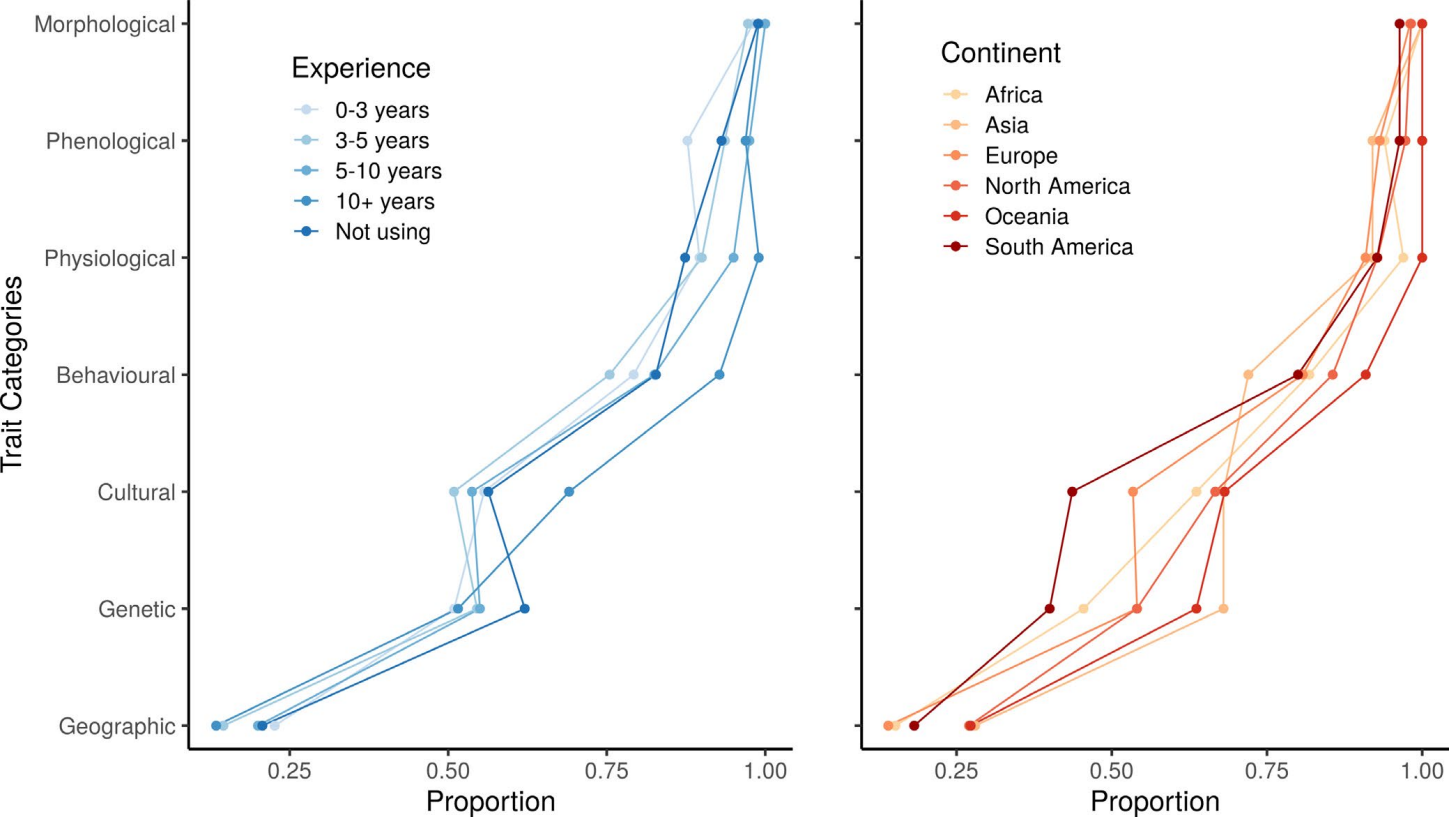
Proportion of respondents in agreement with the different options for Question 1: “The following are acceptable measurements of a biological ‘trait'.” Respondents are grouped according to their research experience (left) and continent in which they perform their research (right). Note the overall consistency among groups in the agreement proportions

When asked about what should be considered a “trait,” respondents agreed in some cases but also showed divided opinions. Ecologists largely agreed that “morphological” (99% agree), “phenological” (94%), “physiological” (92%) and “behavioral” (83%) are acceptable trait categories, whereas “geographic” is not (82% disagree. Figure 1). Within individual variables for these categories, most respondents considered that characteristics, such as “habitat fragmentation” (89%), conservation status (87%), species distribution range (73%), and population density (73%) should not be considered traits. Most respondents considered “photosynthetic rate” (94%), “seed production” (93%), “leaf size” (92%), “desiccation tolerance” (91%), “body mass” (90%) or “body length” (90%) as traits. However, opinions were more heterogeneous regarding the “cultural” (57% agree) and “genetic” (55%) categories, with clear division of opinions for features, such as “home range size” (43%), “population growth rate” (53%), “inbreeding coefficient” (53%), “allele frequency” (55%), “genotype” (56%), or group size (56%).

Disagreement also occurred about the scale at which a “trait” can be defined. While there was consensus about the notion of defining traits at the scale of “individuals” (95%), we found less agreement within individual scales: “organ system” (77%) and “clonal line” (69%). Division among researchers was also clear for larger scales (Supplementary Information 3; Fig. S3.1). When asked about the conditions that a trait must fulfil to be considered a “functional trait,” respondents showed disagreement again, with “affect ecosystem processes” (50% agree), “be related to resource acquisition” (50%) and “define important niche dimensions” (50%) being particularly divisive. We only found agreement on the fact that few researchers expected no conditions should be met (“none” 8%). Surprisingly, about a quarter (23%) of respondents did not consider that functional traits should “affect organism fitness,” even though several of the most widespread definitions state this requirement (e.g., Violle et al., 2007).

In the remaining questions, the respondents were asked to show their degree of agreement with several statements, and as above, we found variation in responses. When asked about their agreement with “A biological “trait” must not be defined by its relation to the environment,” 10.2% of respondents were “unsure,” 38% agreed and 44% disagreed, and 18% had a “neutral” opinion. Opinions were more certain about “A biological ‘trait’ must be heritable” with only 3% respondents being “unsure,” but opinions remained divided with half the respondents disagreeing and 36% agreeing. Finally, 81% of respondents considered that the terms “traits” and “characteristic” could be interchangeable (8% “always,” 37% “most times,” and 36% “sometimes”), whereas 19% did not consider this equivalent meaning as generally correct (8% “never” and 11% “few times”).

**FIGURE 2:**
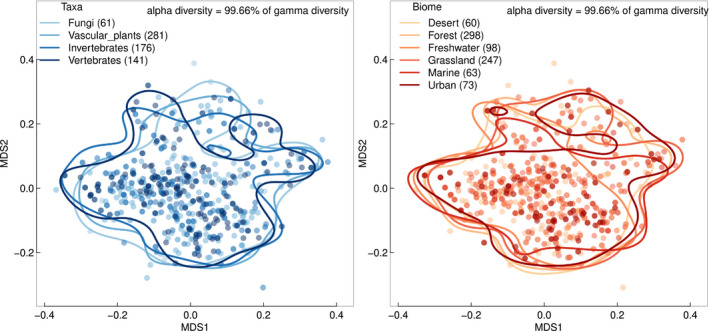
Differences in global opinions among respondents grouped into “communities” based on the primary taxa (left) and biome (right) on which they perform their research. Each point represents a respondent on a nonmetric multidimensional scale analysis based on the pairwise dissimilarity between responses to all the questions. For each community, the lines represent the 95% probabilistic contour. Note the high overlap between the different groups, and the very low proportion of variability in responses explained by the groups (i.e., differences within group members account for >99% of total variability in both cases)

The estimated average Rao value for different subdisciplines did not explain terminology preferences of the research community. Within community, diversity (i.e., alpha diversity) was much higher than between community diversity (i.e., gamma diversity). If variation in responses was associated with communities (e.g., subfield of research) then gamma diversity should be much greater than alpha diversity. Instead, we found that for all features, >99% of the total diversity occurred within communities (e.g., within each subfield of research). For example, respondents working with vascular plants, fungi, or invertebrates were not more likely to have similar responses to each other, showing that contrary to our prediction, personal and professional backgrounds (at least those aspects we studied) do not explain the lack of consistency in the understanding of traits among ecologists (Figure 2).

Overall, our questionnaire revealed a high degree of disagreement over what constitutes a trait that cannot be easily explained by differences in lines of research, studied taxa, subfield, geographical origin, or experience.

## 4 LITERATURE REVIEW ON THE USAGE OF FUNCTIONAL TRAITS IN ECOLOGY

To examine how functional traits are described and presented in peer‐reviewed publications, we undertook a literature review. This review focused on 16 journals where functional trait‐based studies are often published: *American Naturalist*, *AREES*, *Ecography*, *Ecology*, *Ecology Letters*, *Ecological Monographs*, *Global Ecology and Biogeography*, *Functional Ecology*, *Journal of Animal Ecology*, *Journal of Applied Ecology*, *Journal of Ecology*, *Nature Ecology and Evolution*, *Oecologia*, *Oikos*, *PNAS*, and *TREE*. Submissions to these journals were considered to represent expert authors and lead contributions to functional trait ecology research. We searched all articles published in 2018 in which “trait*” appeared in the title, abstract and/or keywords. This resulted in 758 papers, which were randomly assigned to eight co‐authors. Data papers, medical papers, and papers in which “trait” was only used generically, or only in the abstract, were removed, which left 712 papers.

For each of the 712 papers, we recorded the following data: (a) number of times “trait” and “functional trait” were used; (b) whether a definition for these terms was provided; (c) whether trait types from our survey (e.g., genetic, morphological, see Section 2) were mentioned as traits in the paper; and (d) study taxa, biome (including lab or modeling) and continent(s) of study. One author then went through all the papers to double‐check for obvious errors. All eight reviewers were assigned 10 papers that were also reviewed by one or more other co‐authors, thereby allowing us to measure reliability among authors. We estimated reliability based on agreement (e.g., whether or not a trait type was mentioned, or biome included, in a paper) tested by Cohen's Kappa (Cohen, 1960) using the irr R package (Gamer et al., 2019). The mean Cohen's Kappa value among reviewers was 0.652, which can qualitatively be interpreted as a moderate (McHugh, 2012) to substantial (Muñoz & Bangdiwala, 1997) level of agreement in interpretation among co‐authors with respect to paper categorization (see Supplementary Information 4 for full analysis).

## 5 RESULTS OF THE LITERATURE REVIEW

**FIGURE 3:**
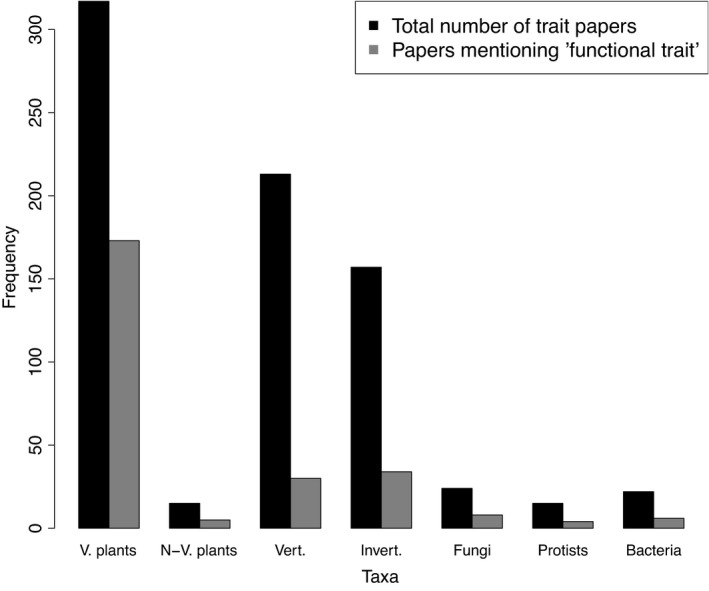
Number of papers out of a total 712 that focus on different taxa (black bars) from the trait literature published in 2018 in 18 selected journals. Frequencies for archaea (2 papers) and viruses (1 paper) are not shown. Papers that include the term “functional trait” in the text are shown in grey bars. Abbreviations: V. plants = vascular plant, N‐V.plants = nonvascular plants, Vert. = vertebrates, Invert. = invertebrates

The 712 papers that fit the criteria for inclusion in our literature review spanned multiple taxa, biomes, and continents of focus. Some papers focused on more than one taxa (73 total, e.g., both vascular plants and vertebrates) or multiple biomes (134, e.g., forests and grasslands; 74 and 35 papers were entirely laboratory and modeling based, respectively). A total of 105 papers (14.7%) focused on more than one continent, or were global in scope (11 were entirely marine focused). In 254 of these papers (35.7%), the term “functional trait” appeared at least once in the body of the text. In the remaining 458 (64.3%), only “trait” was used. Definitions and citations for “functional trait” and “trait” were provided in 31 (4.4%) and 22 (3.1%) papers, respectively. Two additional papers defined “trait” but did not provide a citation. Throughout our analyses, results did not qualitatively differ when only papers that included the term “functional trait” were used in the summary statistics (see Supplementary Information 3). Most papers in our review focused on vascular plants, vertebrates, and/or invertebrates (633, or 88.9% of papers), with only 79 papers (11.1%) studying fungi (24), nonvascular plants (15), protists (15), bacteria (22), archaea (2), or viruses (1). Overall, the specific term “functional trait” (as opposed to just “trait”) was used infrequently in papers that focused on animals whereas it was relatively common in studies of vascular plants (Figure 3). In particular, 54.6% of papers on vascular plants included the term “functional trait,” while percentages were much smaller for nonvascular plants (33%), fungi (33%), vertebrates (14.1%), and invertebrates (21.7%).

**FIGURE 4:**
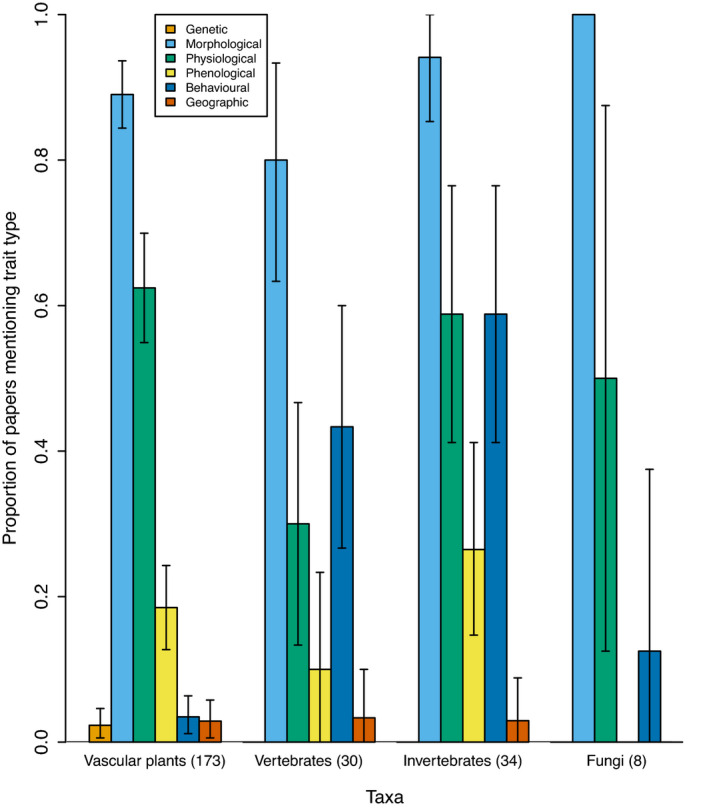
Proportion of papers reviewed focused on different taxa that mention each trait type. We reviewed 712 papers published in 2018 from the functional trait literature covering 16 journals. We present the most commonly studied taxa, nonvascular plants (15), protists (15), bacteria (22), archaea (2), and viruses (1). Papers could include more than one type of taxa and trait type. Error bars show 95% bootstrapped confidence intervals. Bootstrapped confidence intervals were calculated by sampling from binary (YES = 1/NO = 0) vectors with replacement and calculating the distribution of proportions over 10,000 replicates (Manly, 2007)

**FIGURE 5:**
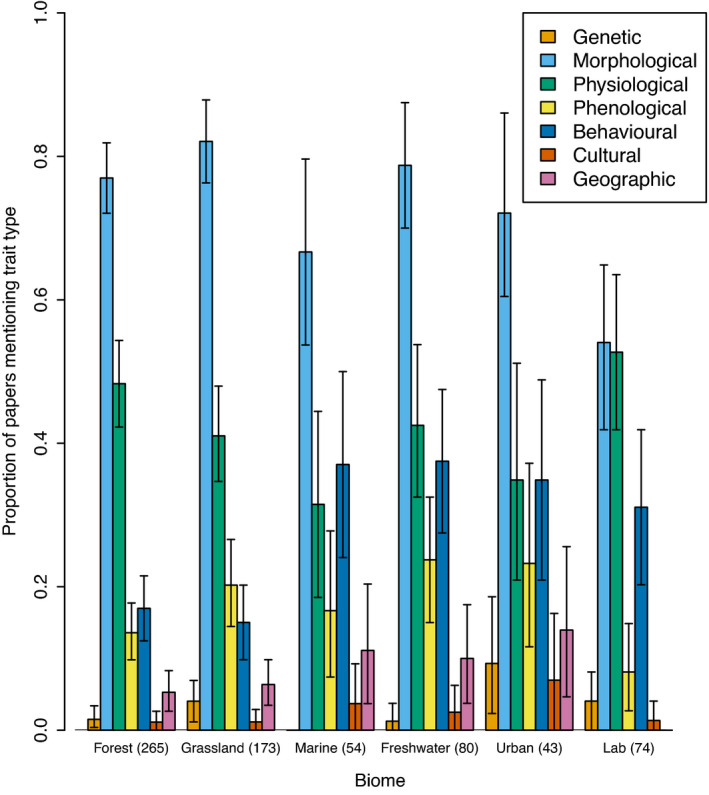
Proportion of mentions for different trait types in the functional trait literature across papers focused on different biomes in 2018. Most papers focused on the six biomes named on the *x*‐axis (number of papers for each biomes are shown in parentheses); biomes not included are benthic (20), desert (37), cropland (17), shrub land (9), alpine (25), modelling (35). Papers might have included more than one biome. Colored bars show different trait types, and the *y*‐axis is the proportion of papers in which each trait type is mentioned for each taxa. Error bars show 95% bootstrapped confidence intervals

Largely, the taxa on which a paper focused had little effect on the types of traits mentioned in the paper. The exception to this observation was for behavioral traits, which were mentioned in 40% of papers focused on vertebrates and invertebrates compared with 7.3% papers on other organisms (Figure 4). There were no noticeable differences across biomes or continents of study in terms of the types of traits mentioned (Figure 5; Supplementary Information 3). Although papers carried out in forest and grassland biomes did mention behavioral traits less frequently than papers carried out in other biomes (Figure 5), this is likely due to the relatively low proportion of papers in these biomes that focused on vertebrates or invertebrates. For example, 133 of the 363 studies (36.6%) that included work in forests or grasslands also included vertebrates or invertebrates, while 210 of the 349 studies (60.2%) in other biomes included vertebrates or invertebrates. Explicit mention of different trait types was consistent across continents, with the exception of studies that only focused on marine organisms, 90.9% of which focused on vertebrates or invertebrates.

Overall, we found little evidence in the literature for major differences in the acknowledgement of different trait types across taxa and regions, although trait papers focusing on plants used the term “functional trait” more often, and trait papers focused on animals were more likely to mention behavioral traits. The latter is unsurprising given behavioral traits tend to be animal‐specific, similar to photosynthetic traits only being measured in plants.

## 6 GENERAL CONCLUSIONS FROM THE SURVEY AND LITERATURE REVIEW

### 6.1 Survey vs. literature review

The survey results suggest that researchers generally conceive traits the same way they are used in the literature. The significant disagreements in trait definition in the survey were unexpectedly not attributable to any particular taxa, biome or continent, and were evenly spread among subdisciplines. The large disagreements in the survey about trait definitions and the conditions necessary for a trait to be considered “functional” would be hard to detect in the literature; few of the studies we reviewed provided definitions or citations of the terms “trait” or “functional trait.” If researchers disagree on the meaning of a term and yet fail to provide definitions when they use it, it could be because they are either unaware that others are using the term trait and functional trait differently or are aware and do not wish to “open that can of worms” in their publications by providing a definition that may be viewed as wrong by reviewers, editors, and readers. Either way, this presents a problem. Publishing papers with unrecognized ambiguous terminology hampers one of the central tenets of functional ecology: that focusing on functional traits enables and enhances comparability between studies and analyses across spatial and organizational scales (McGill et al., 2006; Shipley et al., 2016). Achieving this goal requires clarification and agreement within the wide community studying functional traits on future definitions.

In terms of types of traits, some, like morphological traits are well used in the literature and broadly accepted by the survey respondents. However, other categories that were generally supported as valid in the survey (behavioral, cultural, and genetic) hardly appeared in the reviewed papers. Two possible explanations for this are that plants continue to dominate the trait literature and that morphological traits are the easiest to measure, and thereby the most widely used. Nonmorphological traits may also be under‐explored because they are perceived as being less informative or less widely recognized, therefore studies based on these less used traits (e.g., cultural or genetic) may be more difficult to publish. Whatever the explanation, the outcome is a bias in the literature toward morphological traits despite the fact that many researchers recognized other trait types. By focusing heavily on morphological traits, we may be missing important processes and insights. We cannot fully operationalize trait research across organisms without first directly addressing what constitutes a trait and how they can be used.

### 6.2 Classic definitions vs. current use

It appears that although classic definitions, such as Violle et al. (2007) and McGill et al. (2006) are often used in trait parlance, current opinions (i.e., survey results) of functional traits do not completely align with these. Is this a problem? Potentially, as there seem to be discrepancies among researcher interpretations, meaning that research may not be directly comparable and term ambiguity can lead to misuse of data and findings. Despite available definitions supposedly being applicable to all organisms, it was also found in the survey that researchers perceive their application as flexible depending on study organism. This suggests that despite the universality claimed, currently available definitions are not easily applicable to some organisms such as those without marked individual boundaries (e.g., fungi, clonal mosses, and colony ants). The fact that recent papers are still offering altered definitions of functionality or traits (e.g., Bellwood et al., 2019; Dawson et al., 2019) further supports our claim that current practitioners are unable to make classic definitions work in their field. Unification and clarity in the use of functional trait concepts and terms may have been achieved in plant ecology following the Violle et al. (2007) definition, which is widely accepted and used (e.g., Hortal et al., 2015; Velland, 2016). However, we are still attempting to find universally useful terms applicable beyond plants.

Our survey shows that there are two main aspects of the classic trait definitions over which there is ample disagreement today: that a trait must be heritable and that a trait must be independent from the environment. Violle et al. (2007) have the environmental independence clause in their definition, and Garnier et al. (2015) emphasize that a trait must be heritable. The emphasis on heritability may lead to confusion because, much like the term “trait,” “heritability” is used both as a colloquial term to indicate generic causal relationships attributable to ancestry (as opposed to incidental acquisition during life for reasons entirely unrelated to ancestry), and more accurately as a technical term with a specific meaning in biology (Visscher et al., 2008). In quantitative genetics, heritability is defined as the proportion of phenotypic variance in a population that can be attributed to variation in total (broad‐sense heritability, *H* ^2^) or additive (narrow‐sense heritability, *h* ^2^) genetic values (Visscher et al., 2008). Because these values can and do change over time and across environments at times offering estimates of zero heritability, this would imply that whether or not something is a trait can change depending on where or when it is measured, a less than desirable property. Confusion surrounding the trait independence from its environment clause may also result from differences in interpretation. What is represented by a trait, its definition, can be independent of the environment even if to obtain measurements or values of that trait we must do so within given environmental conditions, which ideally are standardized. So, while traits should be *defined* independently of the environment, they are *measured* under some set of environmental conditions. It is for this reason that trait handbooks (e.g., Perez‐Harguindeguy et al., 2013; Moretti et al., 2017) define standard conditions for measuring traits. For example, growth rate can be defined independently of environmental conditions, but if we want to compare growth rate estimates among different organisms to see which are faster growers, we must compare measurements taken under consistent, reported environmental conditions. Although the opinions of trait researchers are split on these aspects, they are rarely addressed or specified in the literature, resulting in further confusion within trait ecology.

Finally, the plethora of trait definitions to choose from, each with slightly different emphases, caveats and subcriteria, increases the difficulty of clarifying functional trait terms. Violle et al. (2007) and McGill et al. (2006) are often presented as the classic terms and many have adapted these definitions; however, new or adapted definitions are still being created (e.g., Bellwood et al., 2019; Brodribb, 2017; Dawson et al., 2019; Gavel et al., 2016; Schneider et al., 2019). The increasing number of definitions, each adapted for different target organisms, study types, or research goals creates further ambiguity and distances researchers from the goal of using functional traits to study all types of organisms in all areas (Keddy, 1992; Shipley et al., 2016). Further, the disagreements and division shown in the survey surrounding trait definitions and what constitutes a trait are likely brought about or enhanced by the ambiguity of trait terminology terms. It is urgent to clarify these terms if functional trait studies are to be truly cross‐species and intercomparable (Shipley et al., 2016).

## 7 WHERE TO FROM HERE?

We identified the need for clarification of trait‐based terminology and application, but we are far from the first to call for such a standardization (e.g., McGill, 2015; Shipley et al., 2016; Violle et al., 2007). Our results from the survey and literature review suggest that despite more than a decade since the first calls, there is still disagreement among researchers over term meaning and ambiguous application in the literature. On a positive note, contrary to expectations, we found that disagreements among researchers were not due to partitioning within subfields, but evenly spaced across the field. This means that there is no need to break out of subdisciplines or choose one over another, but that broad clarification is needed across the entire community.

True comparability will require a truly universal definition applicable to the diversity of life and conditions on the planet, a difficult order to fill. To start, we recommend studies, at a minimum, state or reference definitions they are practicing for trait terminology. Definitions can provide clarification on a study level with minimal effort on the part of the researcher, and make it easier to compare and draw generalities from research in the future. We also propose a return to the basics, embracing a simplified definition that could satisfy more trait ecologists:

A trait is a measurable characteristic (morphological, phenological, physiological, behavioural, or cultural) of an individual organism that is measured at either the individual or other relevant level of organization.

While traits are more commonly defined and measured at the individual level, our definition allows for needed exceptions: life‐forms in which individuals are difficult to define for measurement purposes (e.g., fungi, coral, mosses; lacking defined individuality) or in which many individuals act as a single unit (e.g., an ant colony; a superorganism, sensu Hölldobler & Wilson, 2009). Within individual traits (e.g., biochemical, cellular or organ system) should be reported at the individual level, thereby adhering to the definition above. For organisms lacking defined individuality, traits are often measured at a set area, which could be termed a “functional unit” (e.g., crustose lichens, Mallen‐Cooper & Eldridge, 2016; mosses, Waite & Sack, 2010), per constrained unit for the organism (e.g., saprotrophic fungi in a deadwood log; Dawson et al., 2019) or clonal organisms that can have a pseudoindividual defined (e.g., ramets in plants, fruit bodies in fungi). These are the three main methods for addressing trait measurement in organisms where it is difficult to determine individuality. We believe these methods are valuable and provide a way of incorporating these organisms into a functional trait framework. However, where these methods are applied, they must be explicitly recognized and defined in the publication. For superorganisms, where more than one individual organism makes up the cluster, colony, family, etc., trait expression and measurement may occur at that level (Hölldobler & Wilson, 2009). In fact, in evolutionary biology, some have argued that organized colonies should be viewed as individuals in their own right (Haber, 2013, but see also Gardner & Grafen, 2009). When studying traits of superorganisms, we recommend measurements of the size/abundance of colonies or families are reported to aid in comparison among groups.

Our definition also removes some of the stumbling blocks we identified above regarding the need for traits to be heritable or independent of the environment. Heritability has been suggested to be fundamental to the definition of “trait,” but we prefer not to include it as retaining heritability excludes cultural traits and traits that, while not technically heritable (due to a lack of underlying genetic variation), are otherwise functionally relevant to questions in trait ecology. Hence, the heritability qualification is typically irrelevant (and rarely, if ever, tested explicitly). For example, plant photosynthetic rates might be highly relevant for investigating community dynamics, even if not all species within a community of interest have variation in the genetic values underlying this trait. We also remove the mention of required independence of environmental conditions because while traits should be defined without reference to the environment, trait values (i.e., measurements) generally require references to environmental conditions. The distinction between a trait and a trait value is subtle and this may be the source of confusion reported in the survey. For example, growth rate is the speed at which an organism increases its mass/size and reporting of measurements should include the temperature/humidity/resources provided/etc. To avoid future confusion, we recommend following Violle et al. (2007) and using “trait value” when referring to measurements (e.g., 20 cm) and “trait” when referring to the concept (e.g., height).

Readers may have also noticed we have not defined “functional traits.” Originally, the distinction between “trait” and “functional trait” was clearer (e.g., McGill et al., 2006; Violle et al., 2007), but with time and “buzz‐word” status, this seems to have changed. We have reviewed the literature and have found that “functional” is often redundant or used in a superfluous way. We believe that this issue stems from the attempt to classify functional traits in a binary way, when functionality is rather a continuous variable. For example, it is extremely difficult to demonstrate that any given trait *does not* have a function at some level, under some set of circumstances, either individually or by interacting with other traits (Pistón et al., 2019; Sobral, 2021). Effectively, this means that all traits can be functional to some degree, so that the “functional trait” is a fuzzy concept. Therefore, we suggest that the term “functional” is not used unless ecologists are making an explicit connection, for example, testing if X function is connected to Y process by measuring both. Traits measured in any given study should be relevant to organizational level or environmental conditions that are the focal point of the study (Fukami et al., 2005; Messier et al., 2010; Shipley et al., 2016). By using “functional” in a more explicit way, that is, only when directly examining the functional fitness of organisms, it highlights studies that address a critical but experimentally difficult knowledge gap.

Further, "trait" is also a common English term, which will continue to be applied in other contexts, and trait ecologists should be aware of this. For instance, "trait" has a generic, common parlance meaning of “a distinguishing quality” or “an inherited characteristic.” There are also alternative uses in the biological sciences; for example, the term “trait” has been operational in evolutionary biology at least since the mid‐20th century, when it was defined by Dobzhansky (1956) as “an aspect of the whole or of a certain portion of the developmental pattern of the organism.” There are also a range of “life history traits” that while measured at the individual level are defined at the population level, which are often termed “traits,” for example, longevity, age at first reproduction, number of offspring, etc. These can be seen as common currency for many comparative ecology studies and may also be useful when amalgamating taxa from very different life‐forms into the same study (Healy et al., 2019). However, according to some definitions, these are not technically traits (McGill, 2015), but they will probably continue to be called “traits” by those that use them, highlighting the need for being explicit in publications.

Finally, it is unlikely that there will be universal acceptance of any one definition. For instance, while morphological, phenological and physiological traits have been considered valid for over a decade (Violle et al., 2007), we also included behavioral and cultural traits as all co‐authors agreed that they should be considered traits, an opinion supported by the majority of ecologists according to our survey. Despite the (narrow) majority in the survey who supported “genetic” traits, in the end, we decided not to include it in our trait definition. The genotype/phenotype distinction is broadly used across other disciplines and not incorporating genetic traits keeps our definition consistent with the classic evolutionary interpretation (Dobzhansky, 1956). Given the large number of definitions and the broad spectrum of applications, finding universally accepted points is impossible. Indeed, this paper was conceived because the authors have different opinions on the finer points of trait usage, which arose when discussing how to apply trait concepts beyond plant organisms.

One of the central tenets of trait theory is that functional traits can bring generalities through use beyond species and across multiple taxa (Kraft et al., 2015; Messier et al., 2010; Shipley et al., 2016); however, combining trait data can prove difficult when crossing between kingdoms (e.g., plants and fungi) or phyla (e.g., insects and mammals). While some traits may be taxa‐dependent, we believe that it is possible to use traits across multiple organisms using the definition provided above and carefully selecting more comparable traits. Traits that are more closely linked with fitness (e.g., growth rates) are the most likely candidates to compare across taxa; however, these are “hard” traits which can be very difficult to measure (Paine et al., 2018). Attempting to put disparate organisms in the same study may require measuring these harder and more "universal” traits. The type of study will determine which traits are most useful, with studies focusing on one clade or life‐form able to use more tailored traits and cross‐taxa/clade studies changing perspective to hard traits.

The current disagreement and ambiguity around trait definitions and terminology hampers generalization within trait ecology and communication between researchers. We have shown that in the research community, there is a lack of consensus surrounding terms that is not attributable to researcher subdiscipline, area, or geographic location. Furthermore, terms are not defined or properly referenced in the literature, adding to the opacity of term use. We recognize that universal acceptance of any one definition is unlikely, but we hope our simplified definition will help to address some of the common issues and provide a framework that can be used to define and work with traits across a much broader range of organisms.

## CONFLICT OF INTEREST

The authors declare no conflicts of interest.

## AUTHOR CONTRIBUTIONS

**Samantha K. Dawson:** Conceptualization (lead); Project administration (lead); Supervision (equal); Writing‐original draft (lead); Writing‐review & editing (lead). **Carlos Pérez Carmona:** Conceptualization (lead); Data curation (equal); Formal analysis (lead); Methodology (equal); Project administration (supporting); Writing‐original draft (equal); Writing‐review & editing (equal). **Manuela González‐Suárez:** Conceptualization (lead); Methodology (supporting); Writing‐original draft (equal); Writing‐review & editing (equal). **Mari Jönsson:** Conceptualization (lead); Data curation (lead); Funding acquisition (equal); Methodology (equal); Project administration (equal); Writing‐original draft (equal); Writing‐review & editing (equal). **Filipe Chichorro:** Data curation (equal); Investigation (supporting); Writing‐original draft (supporting); Writing‐review & editing (supporting). **Max Mallen‐Cooper:** Data curation (equal); Investigation (supporting); Writing‐original draft (supporting); Writing‐review & editing (supporting). **Yolanda Melero:** Data curation (equal); Investigation (supporting); Writing‐original draft (supporting); Writing‐review & editing (supporting). **Helen Moor:** Data curation (equal); Investigation (supporting); Methodology (supporting); Writing‐original draft (supporting); Writing‐review & editing (supporting). **John P. Simaika:** Data curation (equal); Investigation (supporting); Methodology (supporting); Writing‐original draft (supporting); Writing‐review & editing (supporting). **Alexander Bradley Duthie:** Conceptualization (lead); Investigation (equal); Methodology (equal); Project administration (lead); Visualization (equal); Writing‐original draft (equal); Writing‐review & editing (equal).

## Supporting information

Supplementary Material

Supplementary Material

Supplementary Material

Supplementary Material

## Data Availability

All data used in the manuscript are available in supplementary material and on Figshare (https://doi.org/10.6084/m9.figshare.16764217). Survey data are fully anonymized.
