# Supplementary material for: The traits of “trait ecologists”: An analysis of the use of trait and functional trait terminology
Source: Ecol Evol. 2021 Nov 11;11(23):16434–45. doi: 10.1002/ece3.8321 (PMC8668725; doi:10.1002/ece3.8321)
Supplement: Supplementary file 1 — Supplementary Material [file ECE3-11-16434-s002.docx]

Table S1.1: Questions asked in online survey and response options where a limited number were selected:

| **Question** | **Answer options** | | | | | | | |
| --- | --- | --- | --- | --- | --- | --- | --- | --- |
| **Q1:** The following are acceptable categories of a biological “trait” measurements (check all that apply) | • Genetic (e.g. locus heterozygosity)  • Morphological (e.g. organism size)  • Physiological (e.g. respiration rate)  • Phenological (e.g. fruiting duration) | | | | • Behavioural (e.g. activity time)  • Cultural (e.g. bird song dialect)  • Geographic (e.g. population density) | | | |
| **Q2:** A biological “trait” can be defined at the following scales (check all that apply) | • Biochemical  • Cellular | • Within-individual  • Individual | | • Group | | • Population | | • Community |
| **Q3:** A biological “trait” must be defined independently from its relation to the environment | • Strongly agree | • Agree | | • Neutral | | • Disagree | | • Strongly disagree |
| **Q4:** A biological “trait” must be heritable | • Strongly agree | • Agree | | • Neutral | | • Disagree | | • Strongly disagree |
| **Q5:** The terms “trait” and “characteristic” are interchangeable in many circumstances/studies | • Always | • Very often | | • Sometimes | | • Rarely | | • Never |
| **Q6:** The definition of “trait” is flexible depending on study organism | • No  • Sometimes, as long as a definition is clearly stated and motivated by knowledge about the organism  • Yes | | | | | | | |
| **Q7:** A biological trait must fulfil the following conditions to be considered a functional trait (check all that apply) | • Affect population growth rate(s)  • Affect organism fitness  • Affect ecosystem processes  • Relate directly to the individual (e.g. no human-designated categories such as provenance or conservation status) | | | | • Be correlated with vital rates  • Be related to resource acquisition  • Define important niche dimensions  • None of the above | | | |
| **Q8:** The following are NOT examples of functional traits (check all that apply) | • Body length  • Trophic group  • Habitat preference  • Population growth rate  • Leaf size  • Canopy height | | • Body mass  • Diet  • Activity period  • Species distribution range  • Population density | | • Inbreeding coefficient  • Group size  • Photosynthetic rate  • Seed production  • Survival rate  • Home range size | | • Dessication tolerance  • Conservation status  • Genotype  • Allele frequency  • Habitat fragmentation | |

Table S1.2: Details asked of survey respondents for classification into fields

| **Q9:** Which of the following terms describes your research? (check all that apply) | • Conservation Science  • Landscape Ecology  • Ecosystem Ecology  • Behavioural Ecology  • Community Ecology | • Evolutionary Ecology  • Population Ecology  • Population Genetics  • Quantitative Genetics | • Evolutionary Biology  • Molecular Biology  • Computational Biology  • Modelling | • Theory  • Taxonomist  • Physiology  • Other (please specify) |
| --- | --- | --- | --- | --- |
| **Q10:** With which of the following taxa do you (mainly) work? (check all that apply) | • Vascular plants  • Non-vascular plants | • Vertebrates  • Invertebrates | • Fungi  • Protists | • Bacteria  • Other (specify) |
| **Q11:** In which biomes do you (mainly) work? (check all that apply) | • Forests  • Grasslands | • Benthic  • Marine | • Freshwater  • Urban | • Desert  • Other (specify) |
| **Q12:** How long have you been working with trait-based approaches? | • I do not work with trait-based approaches  • Less than 3 years  • 3-5 years | | • 5-10 years  • 10+ years | |
| **Q13:** What is your gender identity | • Male | • Female | • Other | • Prefer not to say |
| **Q14:** To which of the following age cohorts do you belong? | • Prefer not to say  • Under 21  • 21-30 | • 31-40  • 41-50 | • 51-60  • 61-70 | • 71-80  • Over 81 |
| **Q15:** What is the highest academic degree that you have obtained? | • Primary education degree  • Secondary education degree  • Two year post-secondary education degree | | • Bachelor’s degree  • Master’s degree  • Doctorate | |
| **Q16:** If you use fieldwork to obtain your data, what continent do you primarily work on? | • Europe  • North America | • South America  • Africa | • Middle East  • Asia | • Oceania |
| **Q17:** What country is your primary institution based in? |  | | | |
