# Supplementary material for: The traits of “trait ecologists”: An analysis of the use of trait and functional trait terminology
Source: Ecol Evol. 2021 Nov 11;11(23):16434–45. doi: 10.1002/ece3.8321 (PMC8668725; doi:10.1002/ece3.8321)
Supplement: Supplementary file 3 — Supplementary Material [file ECE3-11-16434-s003.docx]

**Supplementary Material 3:**

Additional figures to support manuscript findings.

**Section 3a:** Figures based on data from the survey


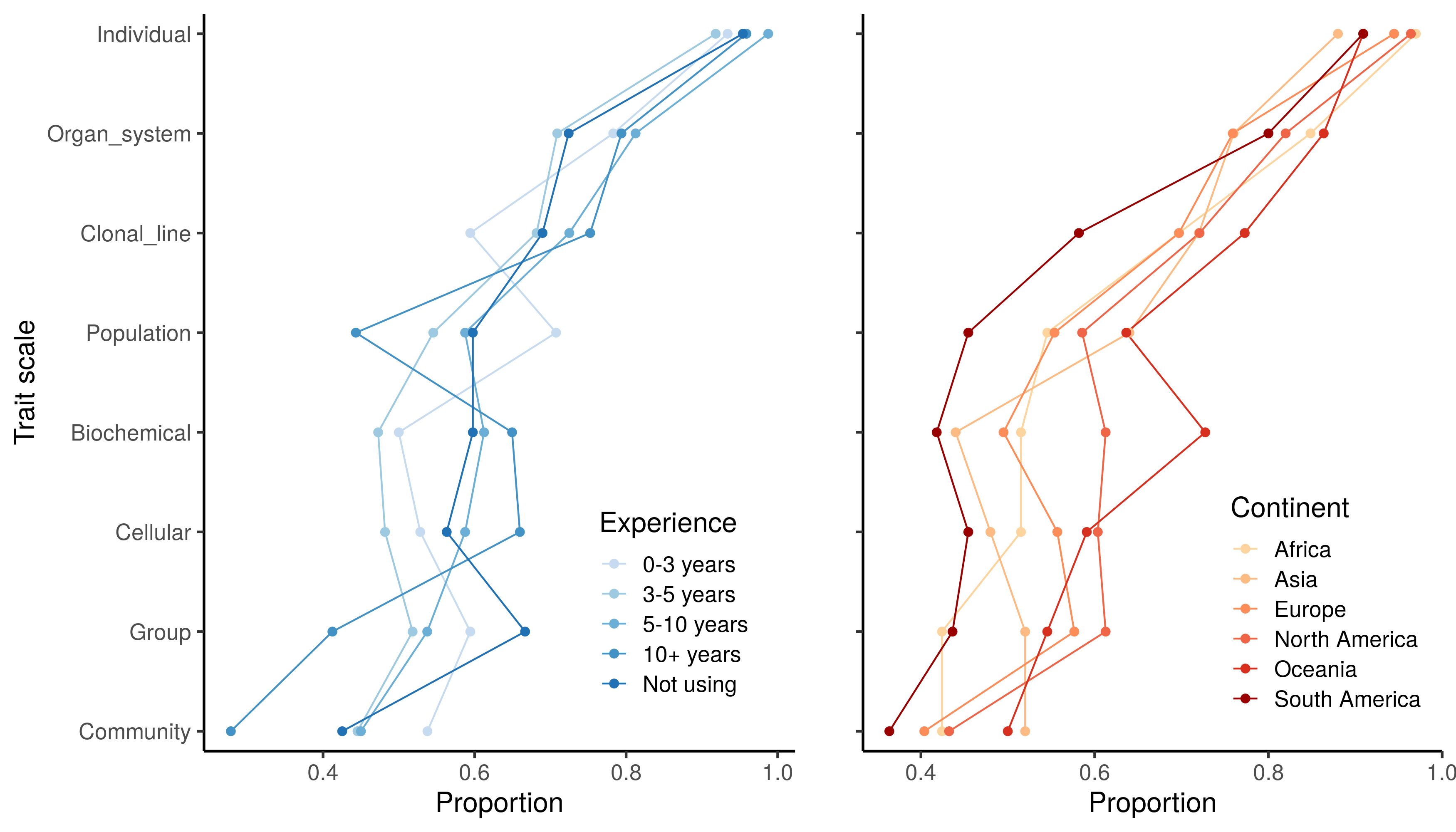


**Figure 3.1.** Proportion of respondents in agreement with the different options for Question 2: A biological “trait” can be defined at the following scales. Respondents here and in following figures are grouped according to their research experience (left) and continent in which they perform their research (right).

**Figure 3.2.** Proportion of respondents in agreement with the different options for Question 3: A biological “trait” must be defined independently from its relation to the environment.


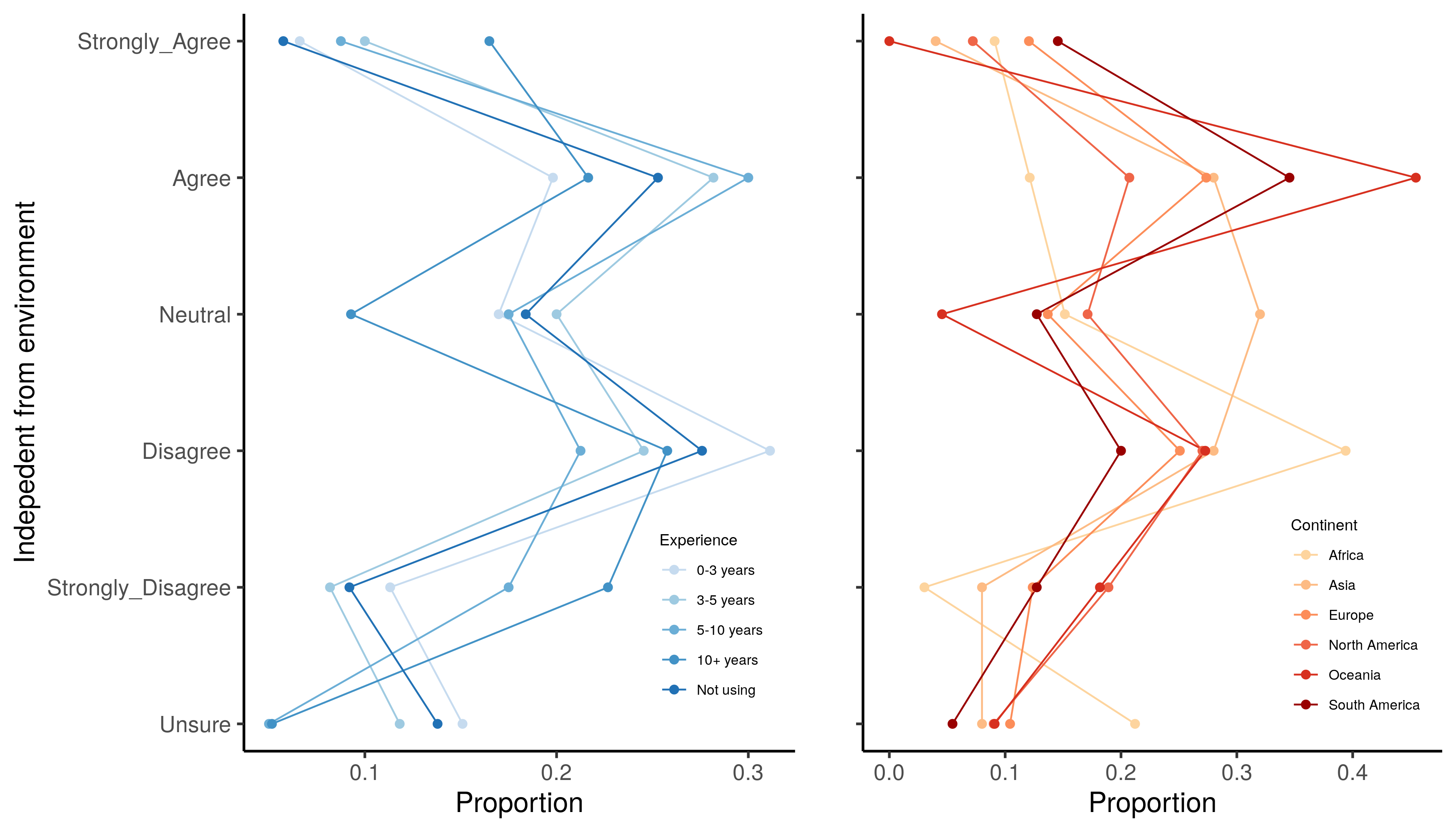

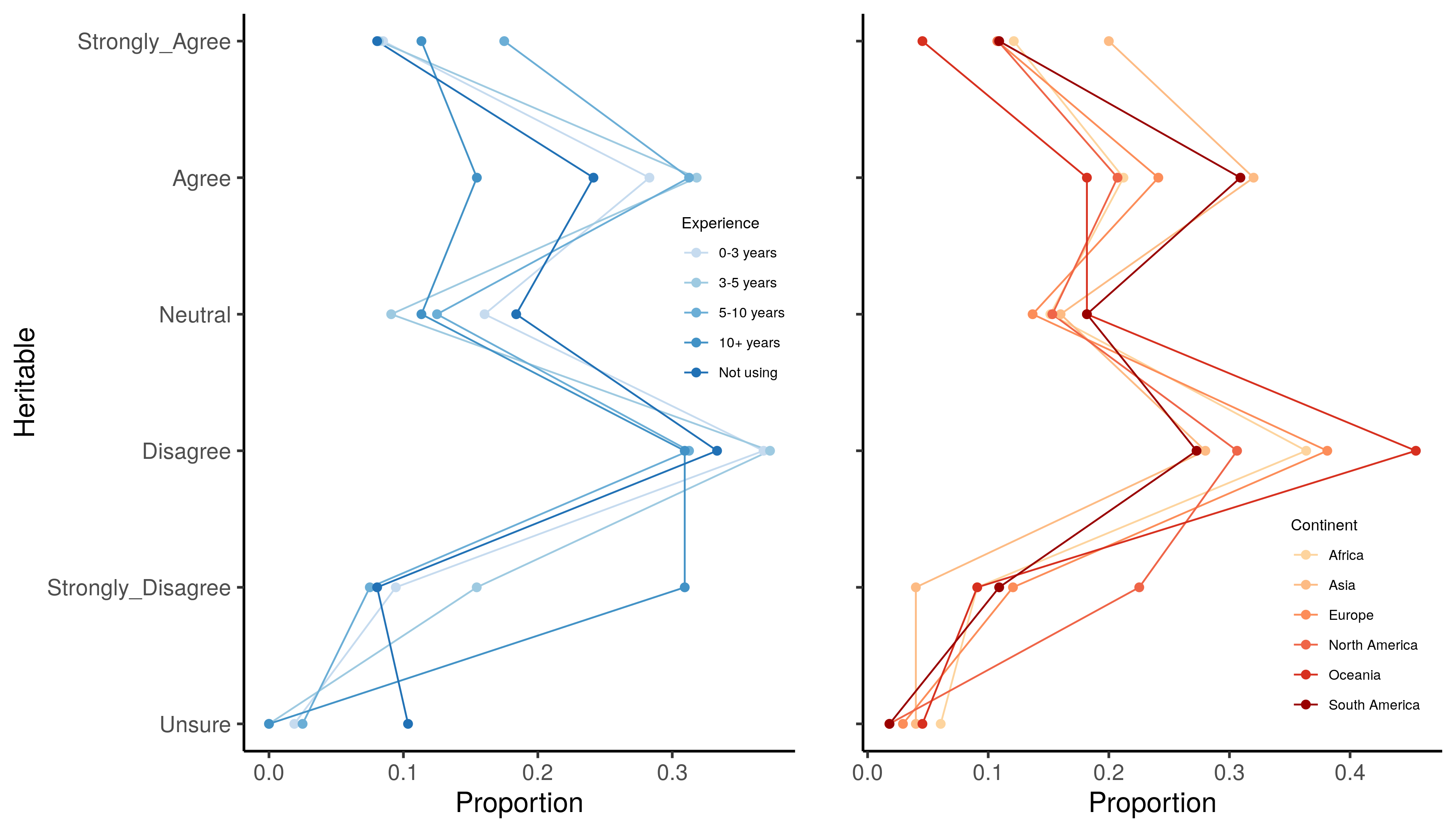


**Figure 3.3.** Proportion of respondents in agreement with the different options for Question 4: A biological “trait” must be heritable


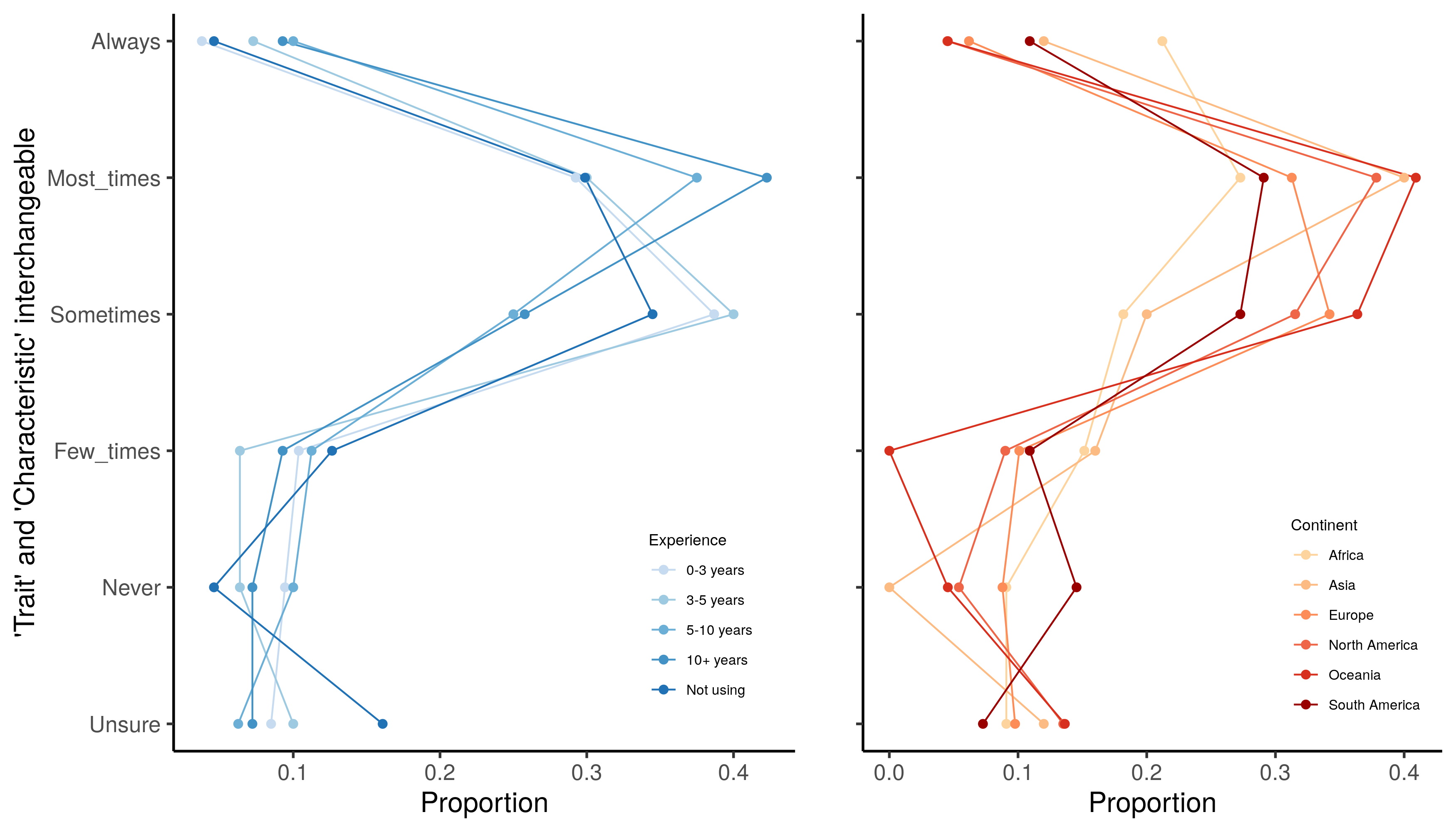


**Figure 3.4.** Proportion of respondents in agreement with the different options for Question 5: The terms “trait” and “characteristic” are interchangeable in many circumstances/studies


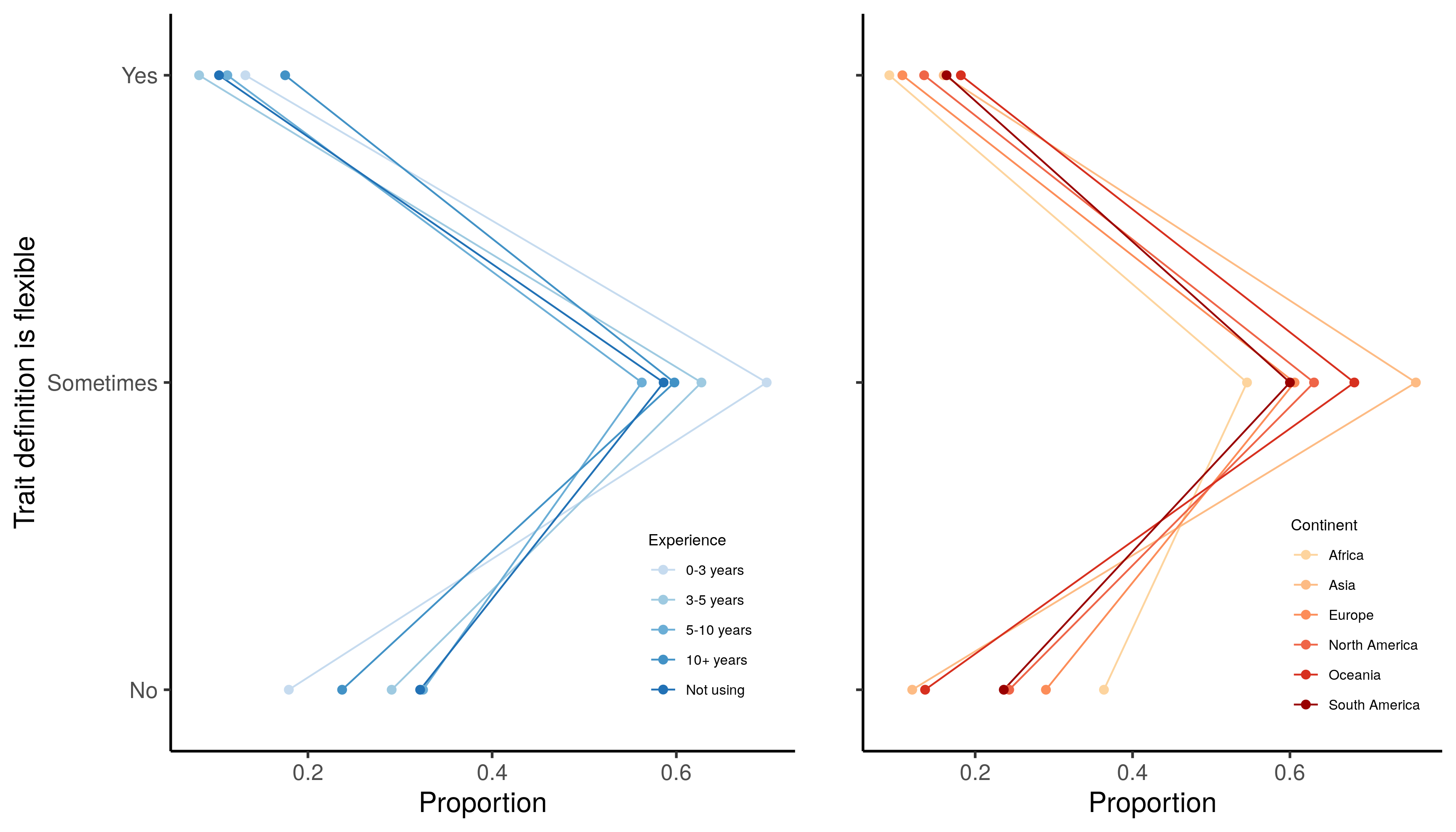


**Figure 3.5.** Proportion of respondents in agreement with the different options for Question 6: The definition of “trait” is flexible depending on study organism


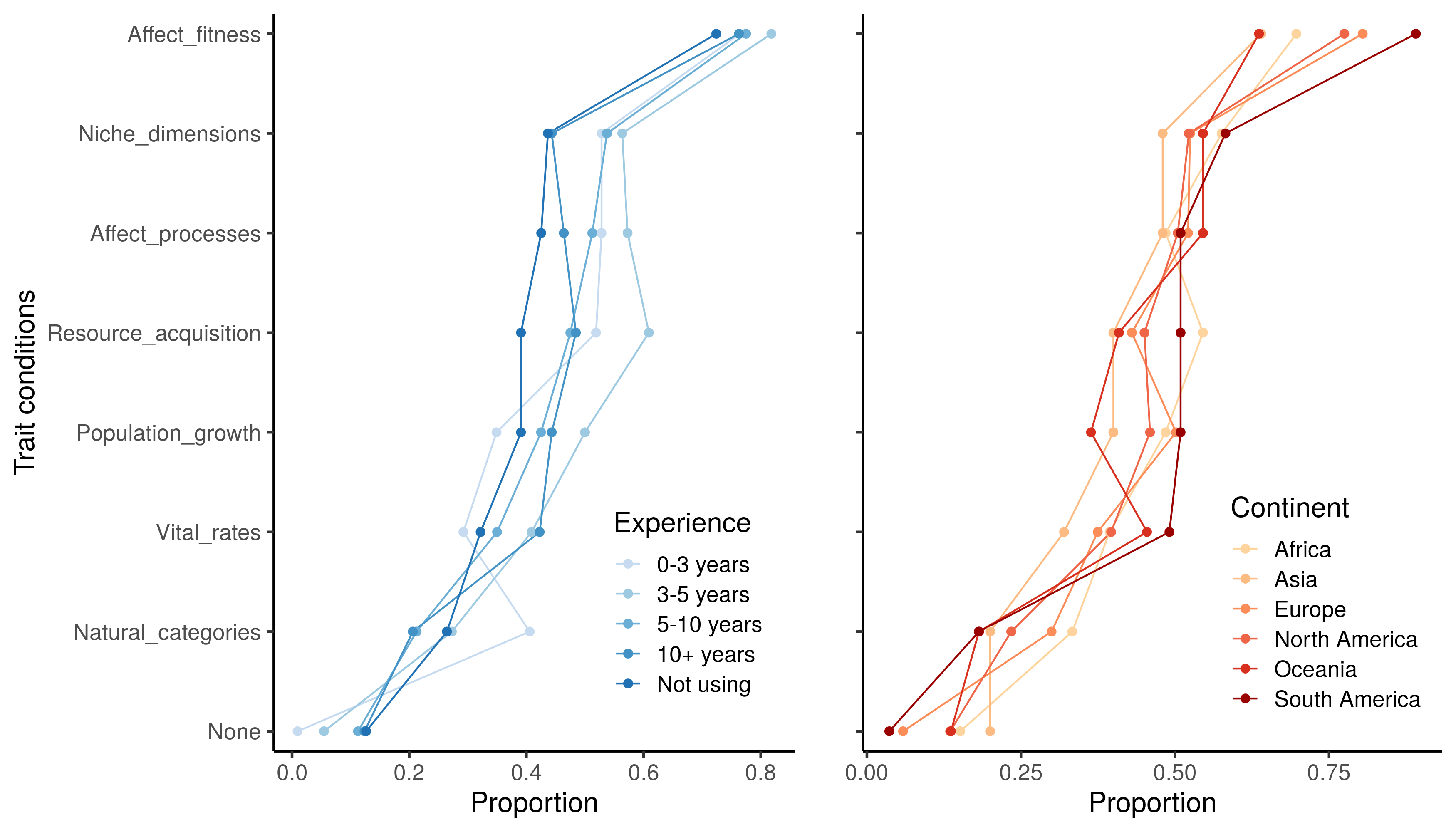


**Figure 3.6.** Proportion of respondents in agreement with the different options for Question 7: A biological trait must fulfil the following conditions to be considered a functional trait


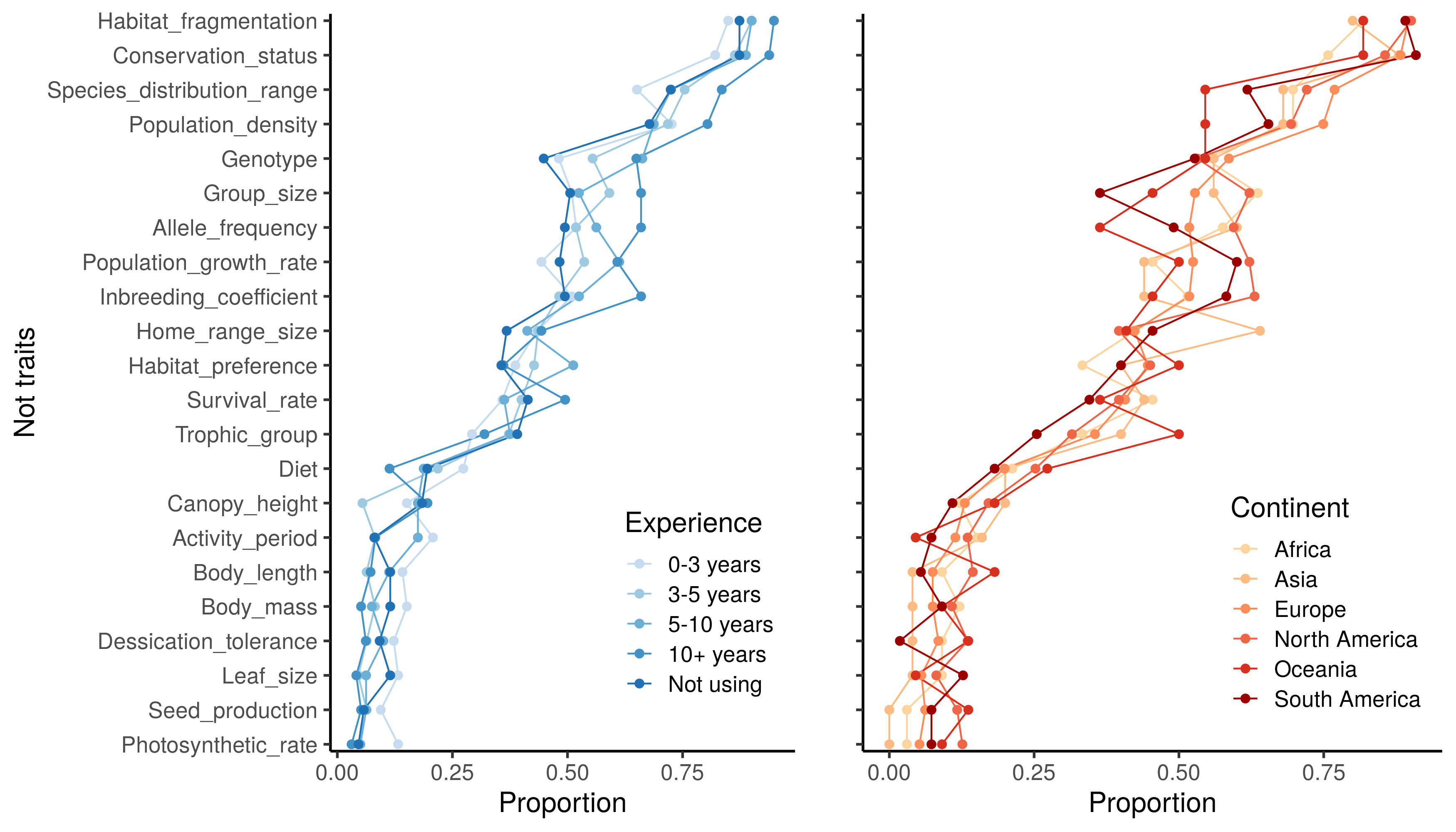


**Figure 3.7.** Proportion of respondents in agreement with the different options for Question 8: The following are NOT examples of functional traits


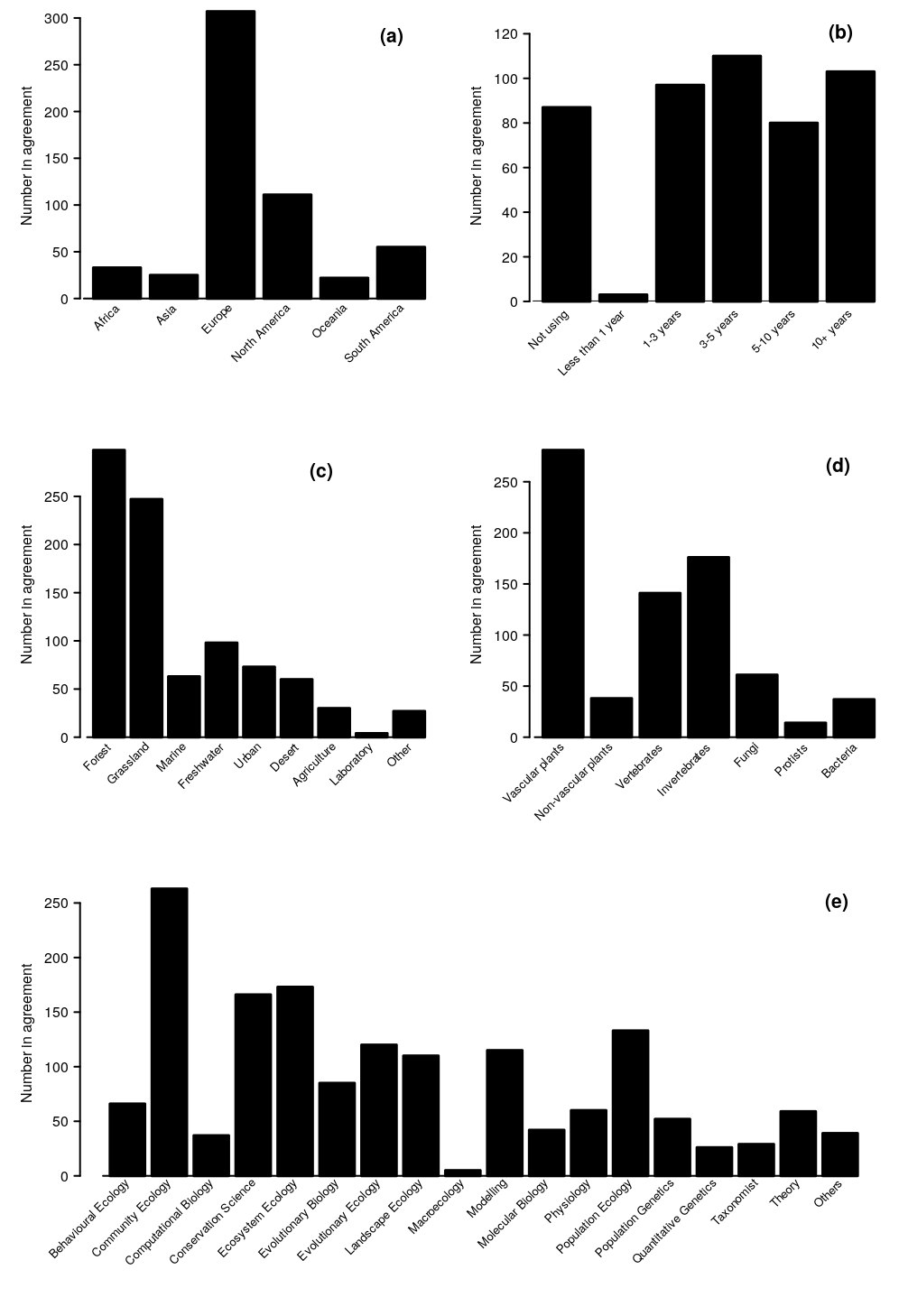


**Figure 3.8:** Number of survey respondents in each of the self-selected categories; (a) continent of data collection, (b) time working with traits, (c) biome of research focus, (d) taxa of interest, (e) term/s that describe research

**Section 3b:** Figures based on data from the literature review


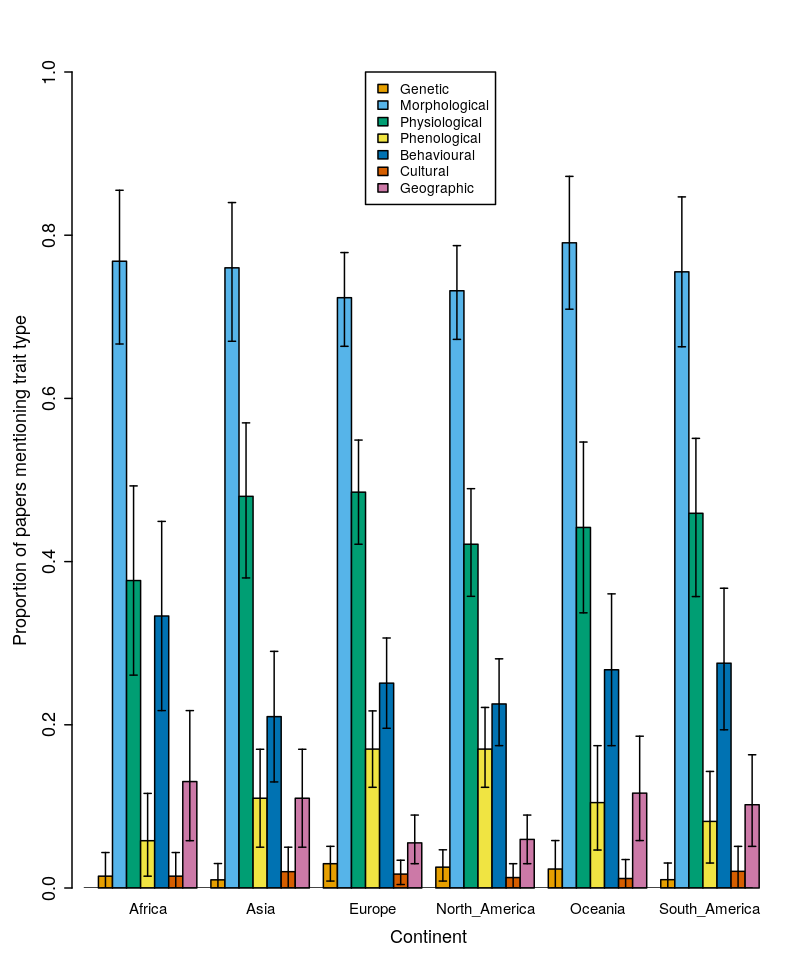


**Figure 3.9.** Proportion of papers that mention trait type organised into continent for the full dataset


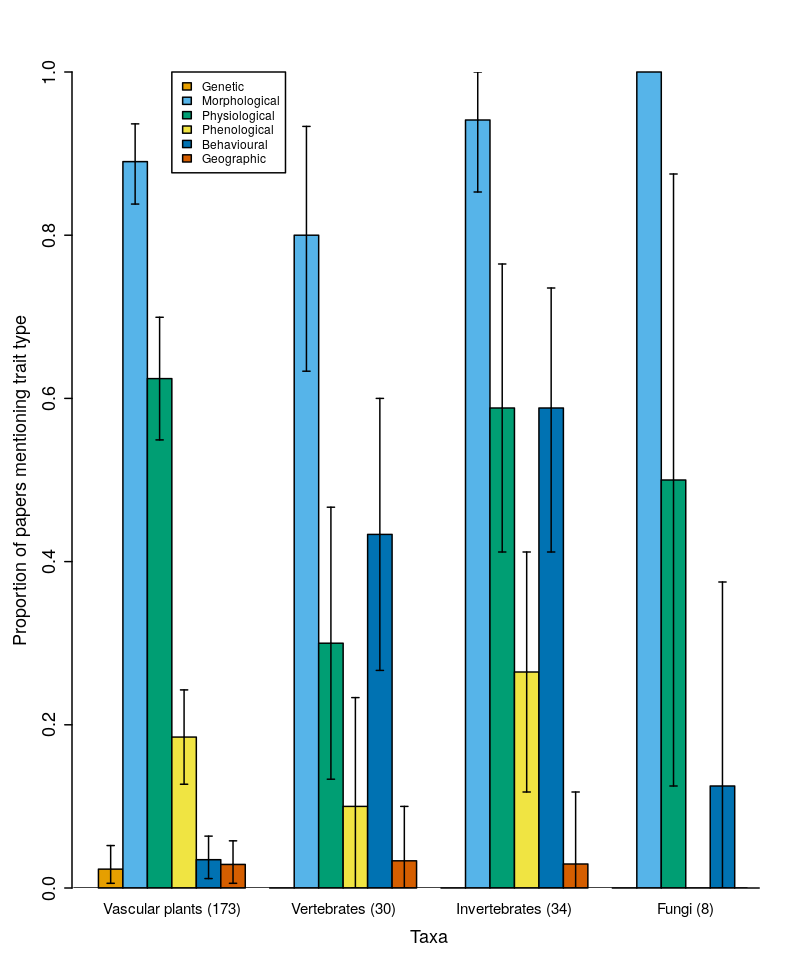


**Figure 3.10.** Proportion of papers that mention trait type organised into organism group for only papers that use the term “functional trait”.


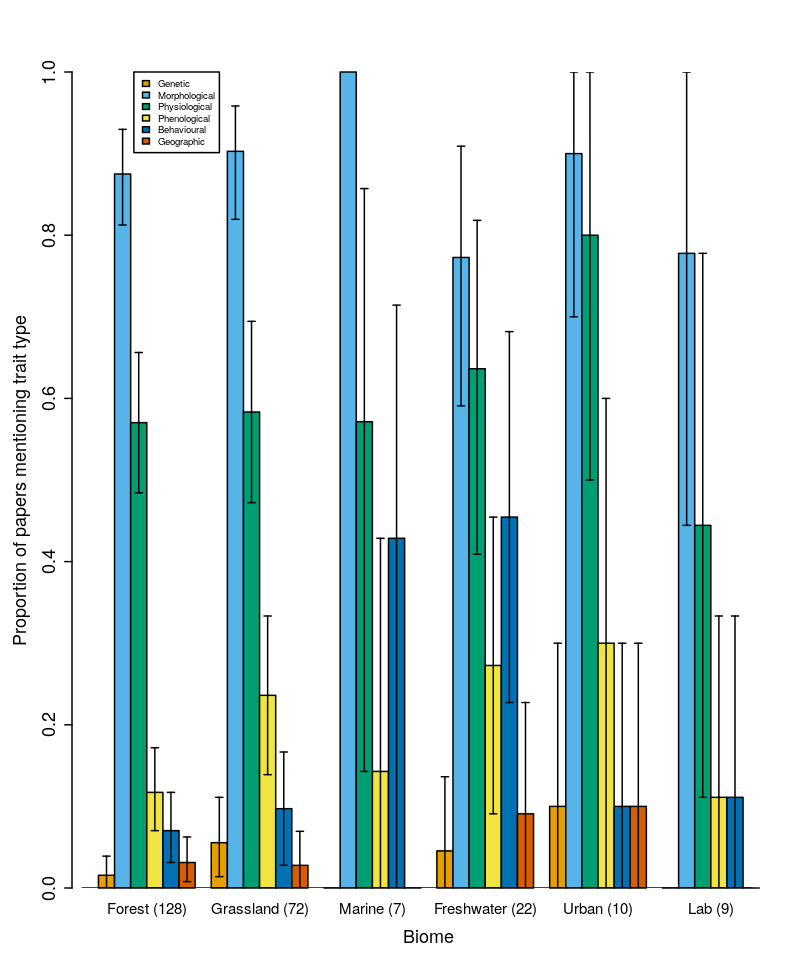


**Figure 3.11.** Proportion of papers that mention trait type organised into study biome for only papers that use the term “functional trait”.


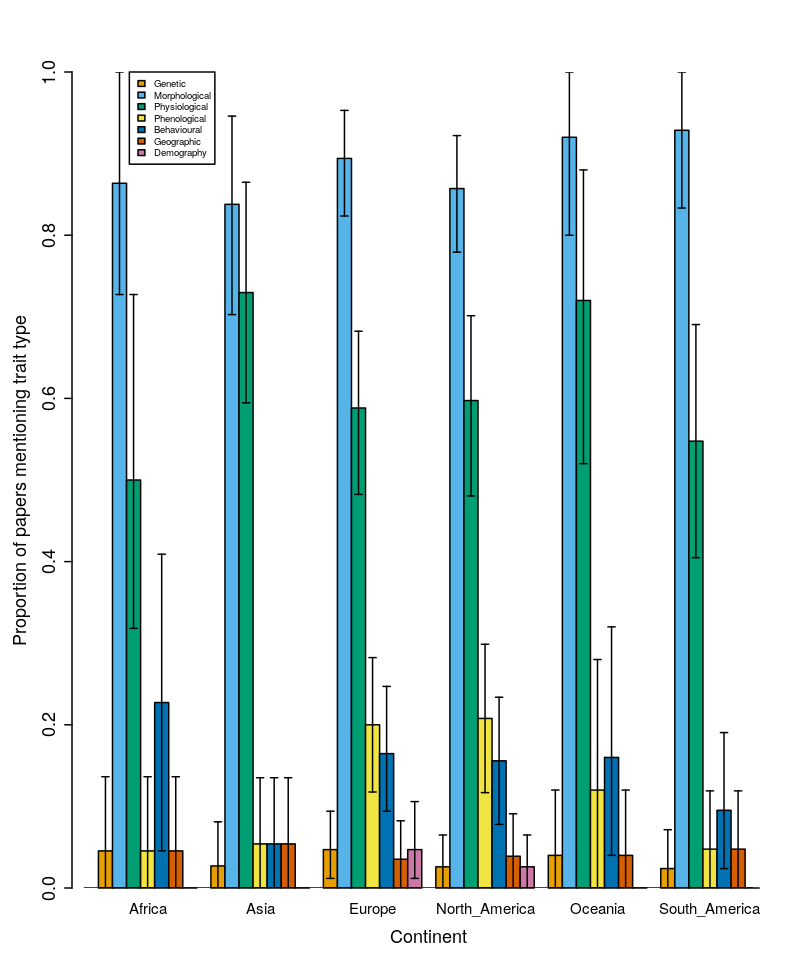


**Figure 3.12.** Proportion of papers that mention trait type organised into continent for only papers that use the term “functional trait”.
