# Supplementary material for: The traits of “trait ecologists”: An analysis of the use of trait and functional trait terminology
Source: Ecol Evol. 2021 Nov 11;11(23):16434–45. doi: 10.1002/ece3.8321 (PMC8668725; doi:10.1002/ece3.8321)
Supplement: Supplementary file 4 — Supplementary Material [file ECE3-11-16434-s004.pdf]

The traits of ‘trait ecologists’: an analysis of the use of trait and functional trait terminology: Supporting Information 4

S. K. Dawson, C. Pérez Carmona, M. Gonzalez-Suarez, M. Jönsson, F. de Carvalho, M. Mallen-Cooper, Y. Melero, H. Moor, J. Simaika, A. B. Duthie

This Supporting Information analyses the agreement among eight reviewers, each of whom reviewed a roughly equal number of a total 712 papers from 16 journals from the year 2018 in which the word ‘trait*’ appeared in the title, abstract, or keywords. This document is also available as in HTML and DOCX form.

Reviewer Data Collection Methodology

Papers chosen for the literature review were placed in a random order and each was randomly assigned to one of eight reviewers. Each reviewer collected key data associated with each of their assigned papers, and made additional comments where appropriate. Key data collected included the following.

- **Reference data:** Journal name, paper DOI
- **Word counts:** Times ‘trait’, ‘functional trait’, and ‘characteristic’ appear in the body of the text (including figure and table legends, but not the abstract or literature cited)
- **Word definitions:** Whether or not ‘trait’ and ‘functional trait’ were explicitly defined, and if so, whether or not references were provided for definitions.
- **Trait mentions** Whether or not a trait within the following category was mentioned (or the category itself was mentioned explicitly as a trait category): Genetic, morphological, physiological, phenological, behavioural, cultural, geographic.
- **Taxa used:** Whether or not the following taxa were a focus of the paper: Vascular plants, non-vascular plants, vertebrates, invertebrates, fungi, protists, bacteria.
- **Biomes used:** Whether or not sampling occurred from the following biomes: Forests, grasslands, benthic, marine, freshwater, urban, desert, laboratory, and in silico.
- **Continent used** Whether or not sampling occurred from the following continent: Africa, Asia, Europe, North America, Oceania, South America.

While some of these data can be collected with certainty (e.g., the paper’s DOI, word counts), other data were uncertain or required some degree of subjectivity. Examples of the latter included whether or not a phrase truly constituted a definition of ‘trait’, whether or not a paper was actually referring to some genetic property as a ‘trait’, or the most appropriate biome to categorise where data were obtained. Because our literature review included eight researchers working independently to collect data on randomly assigned papers, it was possible to assess variation in author interpretation (note that one reviewer checked all data for obvious omissions or errors). We believe that this strengthens our methodology and avoids the potential bias that might have been caused by a single reviewer collecting all data. To investigate reviewer agreement and thereby the reliability of data collection, we used Cohen’s Kappa.

Summary of Cohen's Kappa between reviewers

Cohen's Kappa is a statistic that measures the agreement between two individuals who classify a set of observations (Cohen 1960). It is often used to determine reliability in a clinical setting (McHugh 2012). The statistic accounts for chance agreement between individuals, and takes a value between -1 and 1 with $\kappa > 0$ indicating agreement, $\kappa < 0$ indicating disagreement, and $\kappa = 0$ indicating the expectation of agreement due to chance (McHugh 2012).

We estimated κ between all pairs of eight reviewers, including within-reviewer agreement. To do this, we assigned reviewers to overlapping papers such that each pair of reviewers shared 1-2 papers. We further assigned each reviewer to review at least one paper twice with reviews of the same paper separated by a period of at least four weeks. This allowed an estimate of κ between each reviewer pair, and within reviewers at different points in time. To calculate κ, we used the irr R package (Gamer, Lemon, and Singh 2019). Code to replicate our analysis is available below. Data used for the analysis is can be found at <https://bradduthie.github.io/func_trait_lit_rev/extra_10.csv>. Below is a table of estimates of Cohen's κ between all combinations of eight reviewers.

	Rev_1	Rev_2	Rev_3	Rev_4	Rev_5	Rev_6	Rev_7	Rev_8
Rev_1	1							
Rev_2	0.829	0.763						
Rev_3	0.268	0.828	0.279					
Rev_4	0.516	0.763	0.856	0.501				
Rev_5	0.839	0.691	0.762	0.561	1			
Rev_6	0.693	0.781	0.627	0.89	0.773	0.817		
Rev_7	0.839	0.187	0.668	0.295	0.779	0.712	0.415	
Rev_8	0.681	0.834	0.61	0.642	0.366	0.565	0.402	0.611

Overall, values of κ between reviewers ranged from 0.187 to 0.89. The average value of κ between reviewers was 0.652, while the average value of κ within reviewers was 0.673. While relationship between the magnitude of these values and there interpreted reliability is somewhat subjective, values of $\kappa > 0.6$ can be interpreted as a moderate (McHugh 2012) to substantial (Muñoz and Bangdiwala 1997) level of agreement between observers.

Code to replicate Cohen's Kappa analysis

Below is the code for reproducing the analysis of reviewer agreement, estimating Cohen's Kappa between reviewers.

```r
# First need to read in the 'irr' R package
library(irr);

# Data: https://bradduthie.github.io/func_trait_lit_rev/extra_10.csv
x10  <- read.csv(file = "extra_10.csv", header = TRUE);
udo <- as.character(unique(x10$Article_DOI));
tot <- length(udo);
dat <- NULL;
peo <- NULL;
cou <- 1;
for(i in 1:tot){
    papers    <- x10[x10$Article_DOI == udo[i],];
    if(dim(papers)[1] == 2){
        dat[[cou]]  <- t(as.matrix(papers[,3:(dim(x10)[2] - 1)]));
```

```
        colnames(dat[[cou]]) <- papers[,dim(x10)[2]];
        peo                  <- rbind(peo, colnames(dat[[cou]]));
        cou                  <- cou + 1;
    }
}
# Above code gets through all unique papers

# Now find overlapping answers between two reviewers
peop <- unique(peo[,1]); # Then build a matrix of kappa values
kmat <- matrix(data = 0, nrow = length(peop), ncol = length(peop));
rownames(kmat) <- peop;
colnames(kmat) <- peop;

# Build a list of elements for YES(1)/NO(0) observations
aut_comp <- list();
for(i in 1:dim(kmat)[1]){
    for(j in 1:dim(kmat)[2]){
        aut1 <- peop[i];
        aut2 <- peop[j];
        litm <- paste(aut1,"_",aut2, sep = "");
        for(k in 1:length(dat)){
            authors <- colnames(dat[[k]]);
            if(aut1 %in% authors & aut2 %in% authors & aut1 != aut2){
                if(is.null(aut_comp[[litm]]) == TRUE){
                    aut_comp[[litm]] <- dat[[k]][4:dim(dat[[k]])[1],];
                }else{
                    dat_aut <- colnames(dat[[k]]);
                    if(dat_aut[1] == colnames(aut_comp[[litm]])[1]){
                        col1             <- dat[[k]][4:dim(dat[[k]])[1], 1];
                        col2             <- dat[[k]][4:dim(dat[[k]])[1], 2];
                        add_dat          <- cbind(col1, col2);
                        aut_comp[[litm]] <- rbind(aut_comp[[litm]], add_dat);
                    }else{
                        col1             <- dat[[k]][4:dim(dat[[k]])[1], 2];
                        col2             <- dat[[k]][4:dim(dat[[k]])[1], 1];
                        add_dat          <- cbind(col1, col2);
                        aut_comp[[litm]] <- rbind(aut_comp[[litm]], add_dat);
                    }
                }
            }
            if(aut1 == authors[1] & aut2 == authors[2] & aut1 == aut2){
                add_dat          <- dat[[k]][4:dim(dat[[k]])[1],];
                aut_comp[[litm]] <- rbind(aut_comp[[litm]], add_dat);
            }
        }
    }
}

# Can now calculate Cohen's kappa for each list element in `aut_comp`
for(i in 1:length(aut_comp)){
    kap_val <- kappa2(aut_comp[[i]])$value;
    aut1    <- colnames(aut_comp[[i]])[1];
    aut2    <- colnames(aut_comp[[i]])[2];
```

```
    row     <- which(rownames(kmat) == aut1);
    col     <- which(colnames(kmat) == aut2);
    if(row < col){
        temp <- row;
        row  <- col;
        col  <- temp;
    }
    kmat[row, col] <- kap_val;
}
among_authors  <- mean(kmat[lower.tri(kmat)], na.rm = TRUE);
within_authors <- mean(diag(kmat), na.rm = TRUE);
```

Literature Cited

Cohen, J. A. 1960. "A coefficient of agreement for nominal scales." *Journal of Applied Statistics* 20 (1): 37–46.

Gamer, Matthias, Jim Lemon, and Ian Fellows Puspendra Singh. 2019. *Irr: Various Coefficients of Interrater Reliability and Agreement*. https://CRAN.R-project.org/package=irr.

McHugh, Mary L. 2012. "Lessons in biostatistics interrater reliability: the kappa statistic." *Biochemica Medica* 22 (3): 276–82. https://hrcak.srce.hr/89395.

Muñoz, Sergio R., and Shrikant I. Bangdiwala. 1997. "Interpretation of Kappa and B statistics measures of agreement." *Journal of Applied Statistics* 24 (1): 105–12. https://doi.org/10.1080/02664769723918.
